# Development of a non-human primate model for preclinical research of a novel auditory nerve implant

**DOI:** 10.3389/fnins.2025.1669116

**Published:** 2025-12-10

**Authors:** Inderbir Sondh, Luke A. Johnson, Geoffrey M. Ghose, Aaron Loveland, Lisa Larson, Hubert H. Lim, Meredith E. Adams

**Affiliations:** 1 Department of Biomedical Engineering, University of Minnesota, Minneapolis, MN, United States; 2 Department of Neurology, University of Minnesota, Minneapolis, MN, United States; 3 Department of Neuroscience, University of Minnesota, Minneapolis, MN, United States; 4 Department of Otolaryngology – Head and Neck Surgery, University of Minnesota, Minneapolis, MN, United States; 5 Institute for Translational Neuroscience, University of Minnesota, Minneapolis, MN, United States

**Keywords:** auditory nerve implant, auditory prosthesis, hearing restoration, deafness, cochlear implant, auditory brainstem implant, brain machine interface, neuromodulation

## Abstract

The cochlear implant is a widely available hearing restoration technology that can provide speech understanding in quiet environments. This technology struggles however, in noisy settings or situations involving multiple speakers. The primary cause of these performance limitations is a poor neural interface, in which the bony wall of the cochlea separates the electrode surface from the auditory nerve fibers, thus causing unwanted current spread and non-specific frequency activation. This study utilizes an alternative auditory prosthetic technology (auditory nerve implant, ANI) that enables direct auditory nerve stimulation, which provides a potentially superior neural interface and enables more precise targeting of auditory nerve fibers than traditional cochlear implants. As auditory nerve implants progress towards clinical translation, new implant designs and stimulation strategies will be created. Animal models to efficiently test and iterate through these new designs will be useful for the continued development of ANI technology. We present a viable surgical approach in the non-human primate (rhesus macaque) along with electrophysiological results that demonstrate robust activation of the auditory system at low current levels via intraneural stimulation. Our findings indicate that the rhesus macaque, which possesses an inner ear anatomy more similar to the human compared to other animal models used in the hearing field (e.g., rodents, felines and ferrets), has strong potential as a useful preclinical testbed involving an upright head model for future ANI prototypes and stimulation strategy development.

## 1 Background

For those suffering from moderate to profound hearing loss, hearing restoration can be provided through auditory prosthetic devices such as the cochlear implant (CI). The CI is among the most widely successful neural implants, benefitting more than one million people worldwide (Zeng, 2022). Through electrical stimulation of central and peripheral spiral ganglion cell (SGC) processes (Rattay et al., 2001; Heshmat et al., 2020) [as well as stimulation of inner and outer hair cells (Javel and Shepherd, 2000)], the CI can enable consistent speech perception in quiet settings. The CI performs poorly however, in environments with background noise, multiple speakers, and for music perception (Stickney et al., 2004; Zeng, 2017; Shannon, 2012). Most importantly, settings involving speech-in-noise are still challenging for CI users and thus limit engagement in numerous use cases (e.g., social events, parties, etc.). Music appreciation is also limited among CI users. Most users are only able to perceive rhythm cues, while recognition of melodies or differentiation between musical instruments remains a challenge (Mcdermott, 2004; Philips et al., 2012; Looi et al., 2008). One of the main causes of these limitations is a poor electrode-neuron interface (Zeng et al., 2014; Lim et al., 2017); the bony wall of the cochlea separates the electrode contacts from the auditory neurons. During stimulation, current shunts along the bony wall of the cochlea and is conducted through the perilymph of the scala tympani, leading to broad current spread, spectral smearing, and hampering of selective frequency activation (Friesen et al., 2001; Fu and Nogaki, 2005).

**Figure 1:**
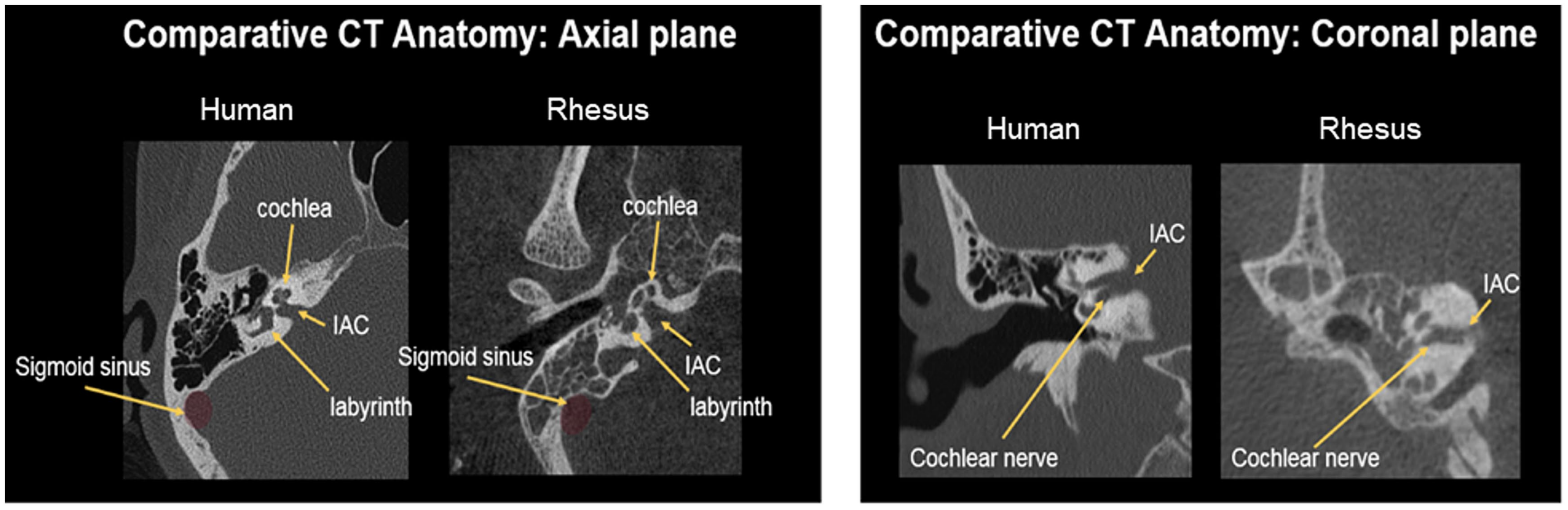
Computed tomography scans of the NHP and human inner ear anatomy. Key structures such as the internal auditory canal (IAC), sigmoid sinus, and cochlea reside in similar relative locations, facilitating translation of the translabyrinthine surgical access procedure. The nerve bundle within the IAC can be readily accessed via drilling through the surrounding labyrinth.

An alternative means of auditory activation, which can help overcome the electrode-neural interface issue, is via intraneural electrical stimulation of the auditory nerve through an auditory nerve implant (ANI) in which electrodes penetrate into the nerve and directly contact the auditory nerve fibers. Through this close contact, the ANI enables specific targeting of the central axons close to the electrode tip. This neural interface is favorable to that of a CI, as it avoids current spread through the cochlear fluid and subsequent wide, non-specific frequency activation. Instead, intraneural stimulation has been shown to exhibit lower activation thresholds and significantly reduced current spread compared with CIs (Middlebrooks and Snyder, 2008; Middlebrooks and Snyder, 2007). The ANI presented in this study features electrode shanks that penetrate the auditory nerve to varying degrees and whose electrode tips contact fiber groups that correspond to different frequency regions. Past studies utilizing a similar design demonstrated selective excitation of fiber subpopulations, suggesting that precise targeting of specific frequency regions is possible with ANIs (Badi et al., 2006). Further electrophysiological and psychoacoustic studies must be performed to determine if the improved frequency targeting ability of ANIs translates to higher fidelity transmission of more complex acoustic patterns than currently possible with CIs. Intraneural stimulation also allows access to low frequency auditory neurons, which are difficult for cochlear implants to access due to their location in the cochlear apex. Activation of low frequency fibers is known to be beneficial for improved speech and music perception (Büchner et al., 2009; Dinçer D’Alessandro et al., 2024). In addition to these electrophysiological advantages, an auditory nerve implant could serve patients who cannot benefit from a CI due to cochlear obstruction, ossification, or malformation. While alternative options for these patients (e.g., auditory brainstem implant) have shown some success in eliciting auditory perceptions, consistent speech understanding remains challenging [please see Lim et al. (2017), Shannon (2012), and Lenarz et al. (2025) for reviews of other auditory prosthetic devices]. Thus, the ANI shows strong potential as an auditory prosthetic technology with possibly improved perceptual performance over the CI. These findings motivate the development and commercialization of ANIs for those suffering from hearing loss. Throughout the translational process, improvements to the ANI technology (e.g., new electrode array designs, novel stimulation algorithms, etc.) will need to be formulated. As novel technologies are developed, continued research in animal models can help to efficiently assess the performance and safety of new ANI devices. Electrophysiological studies could also help in understanding the mechanisms of how electrical stimuli are processed at various stages in the auditory pathway, as done with existing auditory prosthetics (Johnson et al., 2016; Johnson et al., 2017; McCreery et al., 2018). Studies in large animal models, such as non-human primates (NHPs), that share anatomical similarities to humans will be particularly helpful for assessing ANI feasibility. The rhesus macaque is a well-studied animal model that fits this niche. As seen in Figure 1, the macaque exhibits a similar inner ear anatomy as the human, in which the relative locations of key structures such as the internal auditory canal (IAC) and cochlear nerve are comparable. The auditory system of the rhesus macaque has been extensively studied and prior work has characterized its responses to both acoustic and electrical stimulation, allowing for various neurophysiological comparisons for future ANI devices (Kraus et al., 1985; Allen and Starr, 1978; Stahl et al., 2023).

Because of these anatomical similarities, surgical approaches currently used for auditory nerve access in humans can be more easily adapted for the macaque. The translabyrinthine approach is commonly used by skull base surgeons to remove schwannomas and other tumors that have formed on or around the VIIIth cranial nerve (Doig, 1970; Nickele et al., 2012). This approach is well-suited for the goal of nerve implantation, as it allows for full exposure of the internal auditory canal (IAC) and the nerves lying within it. Access to the nerve bundle within the lateral IAC is favorable, as the facial, vestibular, and auditory nerves are known to rotate and become more fused as they progress towards the brainstem (Özdogmus et al., 2004). Thus, clear separation of the auditory nerve from the facial and vestibular nerves is more achievable through a translabyrinthine exposure rather than a traditional retrosigmoid approach that initially exposes a more medial segment of the nerve closer to the brainstem. The surrounding bony anatomy that is exposed also permits multiple options to drill bone grooves, which can be used to anchor the wire bundle of the ANI device. Additionally, the bony backing of the internal auditory canal provides a strong counterforce during insertion of penetrating electrode arrays that helps to drive the electrode shanks into the nerve. A trade-off in this approach is that the vestibular labyrinth must be drilled through and thus vestibular function on the implanted side is sacrificed. The vestibular system is able to compensate for unilateral function loss over time however, through central processes and the remaining vestibular structures on the contralateral side (Dai et al., 2011; Sun et al., 2015). In future studies, we aim to explore alternative surgical approaches (e.g., infracochlear, infralabyrinthine) that better preserve vestibular function by limiting drilling of the vestibular labyrinth.

The current work present results from a series of experiments involving surgical implantation of an auditory nerve implant prototype in the rhesus macaque. The procedure described herein was designed for both chronic and acute implantation studies. While our device design showed robustness during the initial insertion process, we faced challenges with chronic implantation. The array assembly was designed for human use and was relatively large compared to the rhesus macaque anatomy. Sharp bends in the wire bundle were required to maneuver the large array within the small cavity and place electrode sites into the auditory nerve, which created tension in the wire bundle that could shift the electrode sites out of the nerve over time. In addition, the smaller size of the rhesus auditory nerve compared to the human nerve made it difficult to reliably place all electrode sites within the nerve bundle. For future chronic studies, a smaller electrode array is needed that is better tailored to the rhesus anatomy. Due to these technological limitations, the impedance and electrophysiological data presented in this report represent only those collected at the time of initial implantation. Our initial results show promise for our ANI prototype and for the rhesus macaque as a potential preclinical animal model for ANI development. For seven out of eight cases, the majority of electrode sites on the array showed favorable impedance values indicating little to no damage of electrode shanks after initial insertion. In two cases when the electrode array could be stabilized with electrode sites inserted sufficiently within the auditory nerve, stimulation at most sites on the electrode array produced strong activation of downstream auditory structures as evidenced through recordings of the auditory brainstem response (ABR). We also present a method of recording compound action potentials (CAPs) produced by intraneural stimulation of the auditory nerve, in which stimulation and recording take place on the same electrode array, thus providing insight into the local neural activation effects nearby the stimulated electrode sites.

## 2 Methods

### 2.1 Animal care and anesthesia procedures

All animal procedures, tasks, and daily care were carried out in accordance with the National Institute of Health guidelines and approved by the Institutional Animal Care and Use Committee of the University of Minnesota. All animals were housed in standard, commercially-available primate enclosures. Pair-housing was used when possible. Veterinary staff performed daily health checks and served food/water. Environmental enrichment was provided through toys placed in each cage along with movies and music. Nutrient enrichment was provided through fresh vegetables and fruit. Animals were anesthetized with ketamine (5–15 mg/kg, delivered intramuscularly) prior to surgical procedures and remained under anesthesia with isoflurane (inhaled, 1–3%) for the duration of the surgeries. Isoflurane dosage was adjusted periodically based on continuously monitored heart rate, blood oxygenation, body temperature, blood pressure, and respiration rate. Analgesics and antibiotics were administered to animals during and following all surgical procedures for pain management and prevention of infection. At the end of the study period, euthanization was performed through an intravenous barbiturate overdose (>100 mg/kg of sodium pentobarbital).

### 2.2 Electrode array specifications and design motivation

To leverage the tonotopic organization of the auditory nerve and selectively activate specific frequency regions, the electrode array must contact many points within the cross section of the nerve. The Utah slanted electrode array (USEA, Blackrock Neurotech, Salt Lake City, United States), used extensively for intraneural (Badi et al., 2002; George et al., 2020) and cortical (Patel et al., 2023; Sponheim et al., 2021; Normann et al., 1999) stimulation and recording applications, allows for contact of the auditory nerve at different depths via an array of penetrating shanks, thus enabling stimulation of distinct frequency regions. The body of each shank is insulated such that only the tip (metalized with sputtered iridium oxide film) is electrically conductive. By spanning the auditory nerve depth-wise as well as width-wise, this design could allow for a more comprehensive sampling of the frequency space than with, for example, a linear electrode that spans only one track through the cross section of the nerve. Importantly for the translational potential to patients, the USEA has functioned successfully as a chronic nerve implant in humans, such as in studies of the median and ulnar nerves (Clark et al., 2014; Page et al., 2021). These qualities point towards the strong potential for use of the USEA as an ANI.

**Figure 2:**
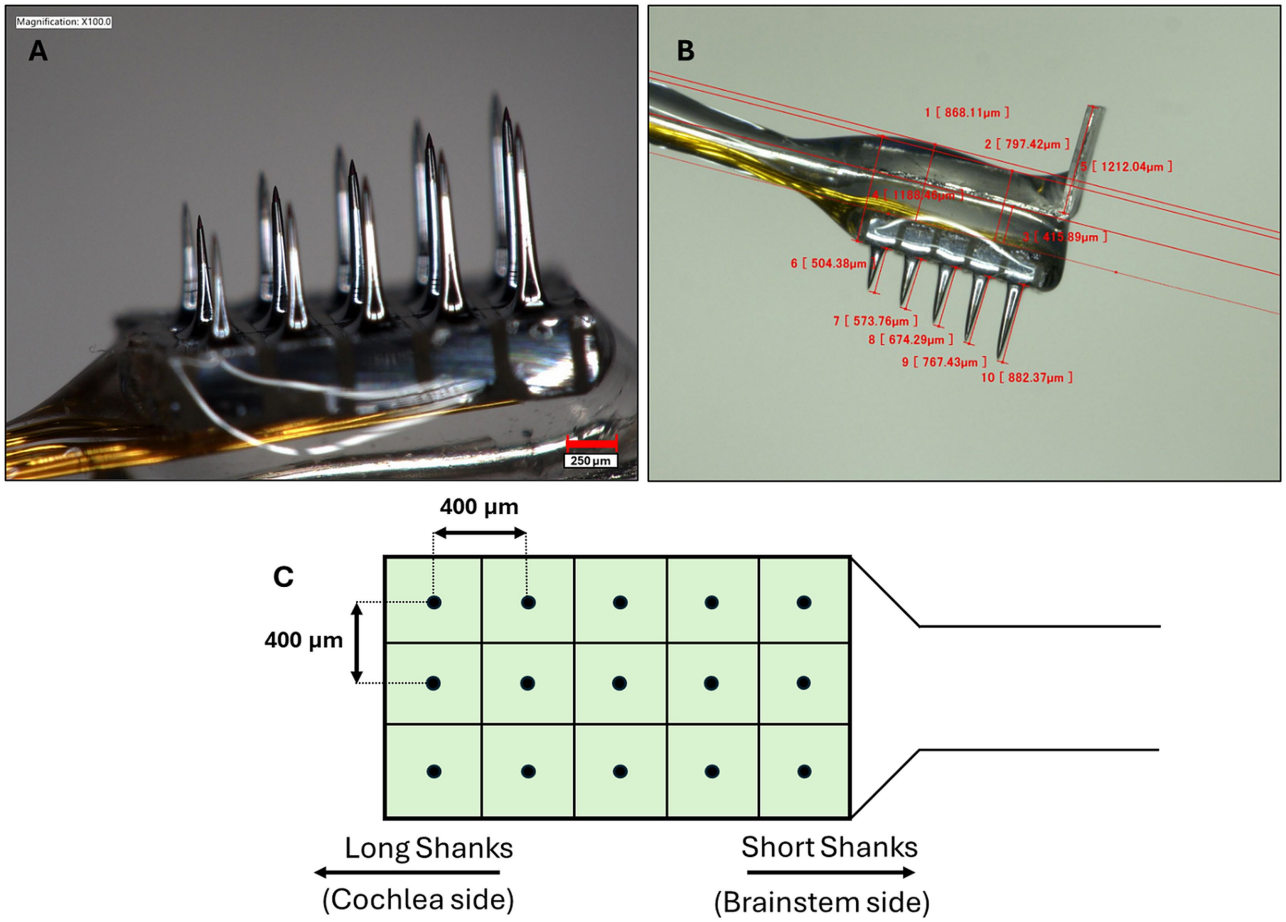
**(A)** The USEA used in this study contains 15 penetrating shanks of varying length, allowing for targeting of different frequency regions of the auditory nerve. The tip of each shank is metallized with sputtered iridium oxide film while the body of the shank is insulated with Parylene C, ensuring that only the electrode tip is electrically active. A 250 μm scale bar is shown in the bottom right corner. **(B)** Shank lengths progress column-wise from 0.5–0.9 mm, with an inter-electrode distance of 400 μm. A platinum fin is provided at the head of the array for handling and manipulation during surgical procedures. **(C)** Overhead schematic of array shank layout. During implantation, the column with the longest shanks is oriented in the direction of the nerve going towards the cochlea while the shortest shanks are oriented towards the brainstem side.

The USEA presented here is a customized version of the standard 96-channel USEA, created specifically to match the size of the auditory nerve in humans. It features a 3×5 rectangular grid of electrodes (Figure 2) and a handling fin for manipulation during surgical implantation. While a higher channel count would allow for finer sampling of the frequency space and thus a potentially larger number of independent frequency channels, a large number of penetrations could also cause damage to the nerve. Future USEA designs will need to investigate how many shanks can be safely inserted into the auditory nerve while balancing the benefit of higher channel counts on improved hearing performance. Higher channel counts could also be achieved by placing multiple electrode sites along the length of the shank in addition to the tip. The length of each shank row ranges from 0.5 mm (shortest) – 0.9 mm (longest) with a step size of 0.1 mm and an inter-electrode distance of 400 μm. Insulated gold alloy wires, packed as a bundled helical coil, were used to connect the electrode shanks to a Cereport connector. Platinum wires were also included in the Cereport connector to serve as recording references or additional grounds. Although this version of the USEA was designed for human use and thus introduced challenges due to its size mismatch with the macaque anatomy, positive outcomes were still achievable during initial implantation. However, future studies involving chronic implantation would benefit from devices with smaller dimensions that are more appropriate for the macaque model, such as a shorter helical wire bundle and an electrode array that is smaller and thus more similar in size to the macaque auditory nerve.

### 2.3 Translabyrinthine surgical approach

**Figure 3:**
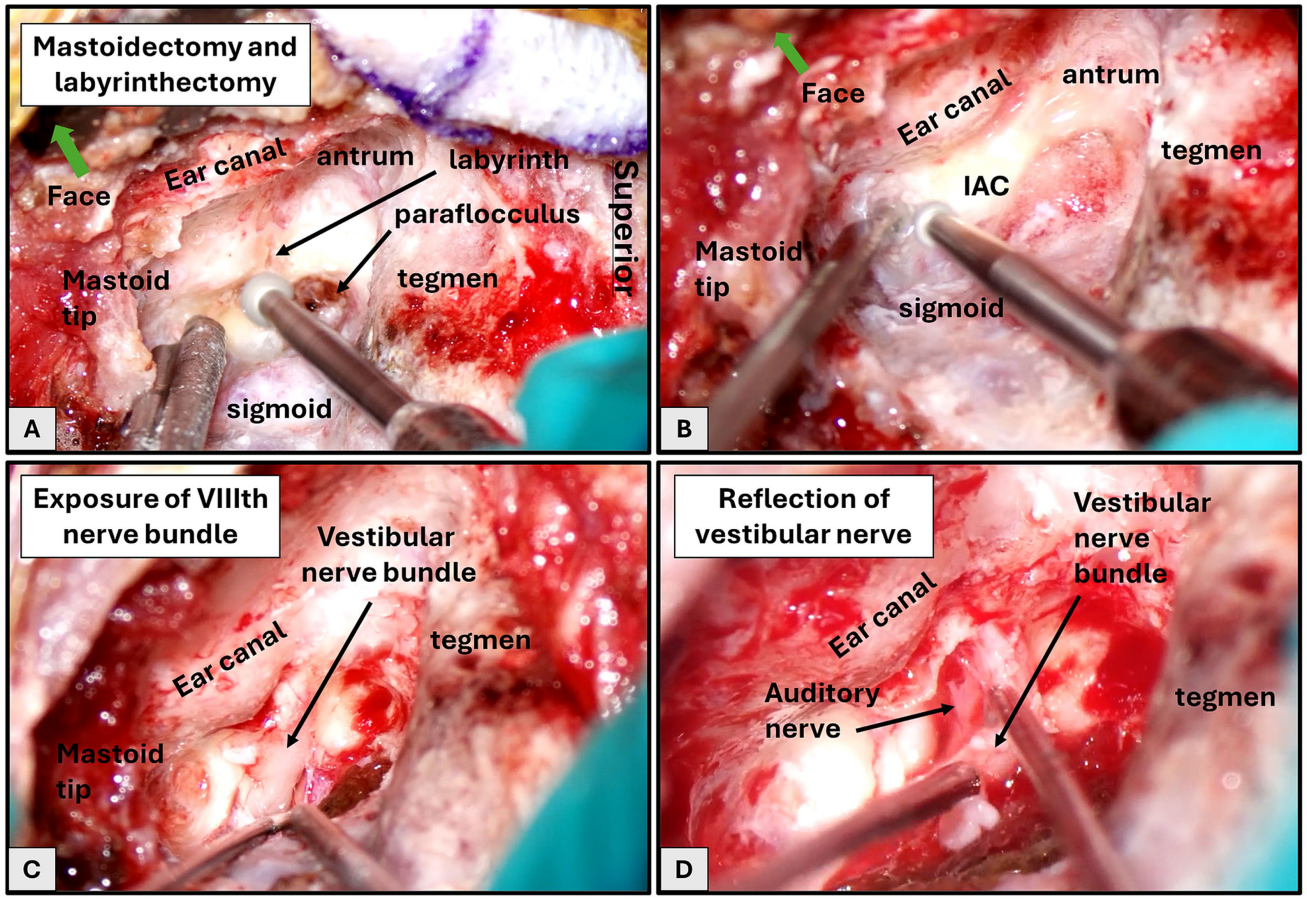
Translabyrinthine approach, left ear. **(A)** Drilling of the mastoidectomy and labyrinthectomy. **(B)** Development of bony troughs inferior and superior to the IAC. **(C)** Initial exposure of the VIIIth nerve bundle within the IAC. **(D)** Sectioning and reflection of the vestibular nerve bundle to show the auditory and facial nerves underneath.

We adapted the translabyrinthine approach for the macaque model as closely as possible to mimic that used in humans. We began the surgery with a C-shaped postauricular incision, staying posterior to the stylomastoid foramen (point of exit of the facial nerve from the mastoid to the parotid gland). This allowed access to the mastoid cortex and identification of the linea temporalis. We then elevated and retracted the post-auricular and nuchal musculature, the temporalis muscle, and the mastoid periosteum. Under surgical microscopy, a translabyrinthine craniotomy was performed via canal wall-up mastoidectomy, whereby the majority of the mastoid air cells were systematically removed with a high speed otologic drill under continuous suction irrigation (Figures 3A,B). Key landmarks were identified and preserved, including the external auditory canal (anterior border), the tegmen mastoideum (superior border, comprised of bone separating the mastoid from the middle cranial fossa), and sigmoid sinus, followed by the mastoid antrum, fossa incudis, lateral semicircular canal, and descending facial nerve. The sigmoid sinus and posterior fossa dura were decompressed as needed, and complete labyrinthectomy was performed. In the rhesus macaque, the cerebellar paraflocculus protrudes through the subarcuate tract; this structure was decompressed to complete the labyrinthectomy.

The internal auditory canal (IAC) was skeletonized along its entire length from the porus acousticus to the fundus. The dura of the IAC was linearly incised and reflected to expose the vestibulocochlear nerve bundle (Figure 3C). The posteriorly positioned vestibular nerve bundle was divided laterally, separating the superior and inferior vestibular nerves from their respective labyrinthine insertion sites, and then reflected medially, exposing the anteriorly positioned auditory nerve bundle as it enters the modiolus of the cochlea and the facial nerve running superior to the auditory nerve within the IAC (Figure 3D).

Vestibular function on the side of the implantation is lost because the vestibular end organs and nerve are sacrificed during the procedure. To reduce undue stress on the animal who must already recover from the implantation surgery, vestibular ablation with gentamicin was performed weeks before the surgery to achieve pre-surgical compensation to single-sided vestibular loss. Prior literature has demonstrated that animals recover well from this procedure and consistently compensate for the vestibular deficit (Dai et al., 2011; Sun et al., 2015). Two intratympanic administrations of gentamicin [~0.5 mL of buffered solution (26.7 mg/mL) via 27-gauge needle were performed], spaced 2–3 weeks apart.

**Figure 4:**
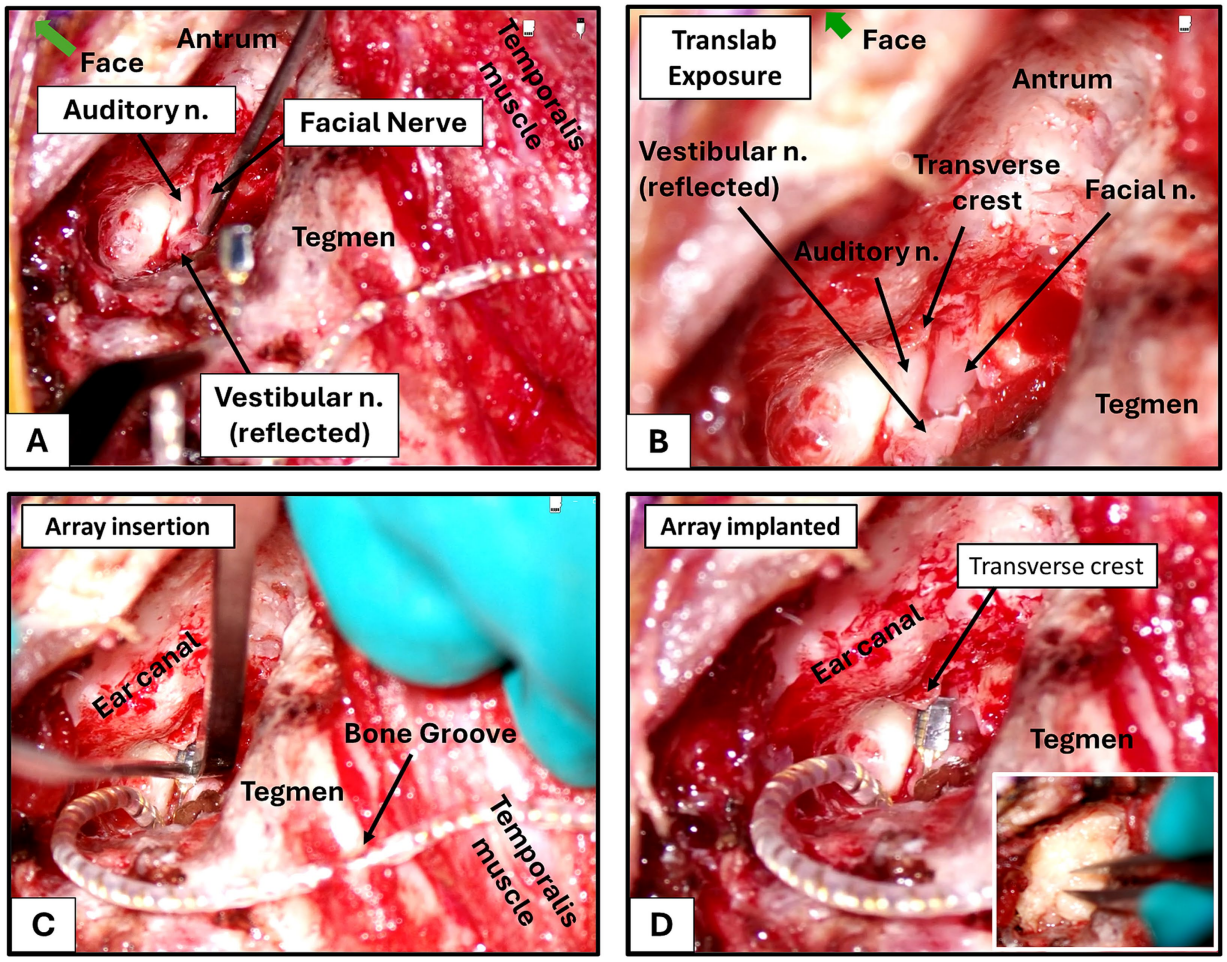
Implantation of electrode array into auditory nerve (left ear). **(A)** Fully exposed auditory and facial nerve within the IAC. The vestibular nerve at this stage has been transsected and reflected medially. **(B)** Identification of key anatomical landmarks prior to insertion. **(C)** Insertion of the array using forceps and osteotome. The array is gently pushed into the nerve until the backing of the IAC is felt, confirming that the longest electrode shanks have penetrated fully. **(D)** Fully implanted USEA. The array is positioned medial to a bony crest to help keep it within the nerve and counteract cerebrospinal fluid pulsations. The remaining loose portions of the wire bundle are firmly fixed into a 0.8 mm bone groove drilled in the temporal bone. Inset shows filling of the space with a fat graft harvested from the abdomen area following final fixation of the wire bundle.

In preparation for electrode implantation and cable fixation, an 0.8 mm diameter bone groove was drilled in the linea temporalis at the posterior aspect of the mastoid cavity. The wire bundle of the ANI device was placed securely into the groove to minimize movement after implantation. The size of the groove allowed for a snug fit of the wire bundle. The exact location of the groove varied depending on individual anatomy. (Figures 4A,B) shows the full surgical exposure of the auditory and facial nerves. The electrode array was positioned underneath a bony transverse crest before being fully inserted using fine tweezers and a surgical osteotome (Figure 4C). In past intraneural (Thomas et al., 2022) or cortical applications (Patel et al., 2023; Rousche and Normann, 1992) of the Utah array, a pneumatic insertion system was used to drive the electrode array into the neural target. Due to its proven efficacy in these other experimental settings, we tested a pneumatic inserter in preliminary cadaver experiments but deemed it ineffective for fully and consistently inserting the array, due to the limited cavity space for proper manipulation and alignment of the inserter trajectory with the array positioning on the nerve. Also, if the tip of the insertion wand was not positioned completely flush with the back of the array, the pneumatic insertion resulted in uneven penetration where shanks on one side of the array penetrated more fully than others. We found that manual insertion with forceps and application of gentle pressure to the array with a small curved osteotome allowed for a more controlled insertion process, as force could be more easily and evenly distributed across the entire back of the array. Thus, we elected to use manual insertion over pneumatic insertion for all implantations reported in this study. The array was placed adjacent to the transverse crest at the lateral IAC, using this structure for stability. Knitted oxidized regenerated cellulose was placed over the implant and IAC. An abdominal fat graft was used to fill the mastoid cavity, providing medial pressure on the array and counteracting cerebrospinal fluid pulsations which could otherwise dislodge the array over time (Figure 4D). This fat graft also helped to prevent cerebrospinal fluid leak. The antrum was sealed with soft tissue and mastoid air cells obliterated with bonewax. A watertight, multilayered closure of the mastoid periosteum, musculature, and skin was performed, and the surgical site was prepped for electrophysiological recordings.

### 2.4 Impedance measurements

Electrode impedance at each electrode site was measured using an IMP-2A-MC 18 channel impedance meter (MicroProbes, Gaithersburg, MD). A 1 kHz sine wave was used as the probe signal, with test currents ranging from 0.24 μA–2.4 μA depending on the impedance range being tested. One site on the USEA was set as the active site, while the base plate of the Neuroport connector served as the return electrode. Each electrode was tested individually, using a physical switch on the impedance meter to cycle through electrodes on the USEA. Based on prior characterization of the USEA in similar settings for other neural targets and clinical applications (Duncan et al., 2019; George et al., 2020) and compliance limits of our stimulator, values of impedance between the range of 10–120 kΩ were used to indicate sites suitable for stimulation. Sites with higher impedances could still be usable for stimulation applications but would require greater compliance voltages to drive enough current through the sites and would also result in higher power and battery requirements for a future neural prosthesis.

### 2.5 Auditory brainstem responses

The ABR is a well-characterized electrophysiological measurement used to assess activation of auditory brain structures from the periphery to the brainstem and has been recorded in the rhesus macaque in prior studies using acoustic stimuli (Kraus et al., 1985; Fria et al., 1982; Laughlin et al., 1999), thus providing reference points for typical response characteristics. For the current study, ABRs were recorded in response to both electrical stimulation via the USEA and acoustic stimulation through a MF1 multi-field speaker (Tucker-Davis Technologies, Alachua, FL) in open-field configuration. Acoustic ABRs were collected to verify healthy hearing in the NHPs prior to electrode implantation. The acoustic stimuli consisted of 10 ms white noise bursts with a 0.5 ms linear ramping. Sound intensity levels ranged from 36 to 76 dB SPL. The electrical stimuli were single, charge-balanced biphasic pulses with 100 μs per phase and an interphase gap of 60 μs. Current levels ranged from 1 to 140 μA. The Cerestim R96 Microstimulator (Blackrock Neurotech, Salt Lake City, UT) was used to generate and deliver all electrical stimuli. Responses to stimulation were recorded using needle electrodes placed at the mastoid (active), vertex (reference) and neck (ground) areas. The needle electrodes interfaced with a RA4L1 low impedance headstage (Tucker-Davis Technologies, Alachua, FL). Up to 1,000 trials of each stimulus setting were collected and averaged together to obtain the final response. Data were band-pass filtered between 300 and 3,000 Hz (4^th^ order Butterworth filter). Notch filters were also employed (centered at 1 kHz, 2 kHz, and 3 kHz) to remove noise generated by the stimulator. Custom MATLAB scripts were employed to control the stimulation and recording workflow and to analyze and plot responess in real time.

### 2.6 Compound action potentials

The electrically-evoked compound action potential (eCAP) captures the summed activity of a population of neurons (He et al., 2017). The eCAP has been used extensively in the auditory research field to measure activation properties of the auditory nerve in response to electrical stimulation (Hughes et al., 2012; Brown et al., 1990; He et al., 2016). The eCAP was measured in this study through stimulation of the auditory nerve at one electrode site and simultaneous recording of the eCAP at another site on the electrode array. All eCAP recordings were performed through a clinically approved system (MaxBox, MED-EL, Innsbruck, Austria). A Sonnet stimulator was used to deliver all electrical stimuli. A total of 100 trials were performed, lowpass filtered (cutoff 18 kHz), and averaged together to obtain the eCAP response.

To verify that the observed eCAP response was not due to stimulation artifact or activation of other nearby tissue or neural structures, two standard procedures were performed: Firstly, the amplitude growth function (AGF) was determined, which tracks the magnitude of the eCAP response as the total delivered charge is increased. In the case of traditional scala tympani stimulation using a CI, the AGF is known to follow a sigmoid trend (Brochier et al., 2021), in which increasingly higher current levels eventually saturate the eCAP response (indicating a maximal recruitment of auditory neurons) and the amplitude ceases to increase. Cases in which the amplitude continues to grow at higher current levels (e.g., >100 μA) could indicate contamination of the response with electrical artifact or activation of non-auditory neural structures. Secondly, the recovery function was calculated, in which a forward masking stimulation paradigm was employed (Hey et al., 2017). The stimulation amplitude was first set to a current level that gave a robust (higher than threshold) eCAP, as determined via the AGF described above. Two stimulation pulses (a masker and a probe) were then delivered in quick succession on the same stimulating electrode, with the gap in between pulses set at a variable masker-probe interval (MPI). The eCAP was then recorded after the 2^nd^ (probe) stimulation pulse. For a true neural response, the eCAP amplitude is known to decrease as the MPI is decreased due to the refractory period of the auditory nerve neurons. If the MPI is critically low, almost all auditory neurons are still in their refractory phase, resulting in little to no eCAP response.

## 3 Results

### 3.1 Translabyrinthine surgical approach ANI array insertion findings

In total, eight nerve exposures were performed across four NHPs. Each NHP was implanted chronically on one side; at the end of the study period, an acute implantation was performed on the contralateral side during a terminal experiment. The data presented here are solely from initial and acute implantations of the array for each NHP. During all chronic implantations however, the implant was well-tolerated and no significant irritation or infection occurred in the weeks following the initial surgery. Vestibular deficits were compensated successfully and the overall behavior of the subjects returned to normal within 1–2 weeks. No observable facial paresis was seen, indicating the facial nerve was not damaged during exposure or implantation of the auditory nerve and there were no cerebrospinal fluid leaks or meningitis.

**Figure 5:**
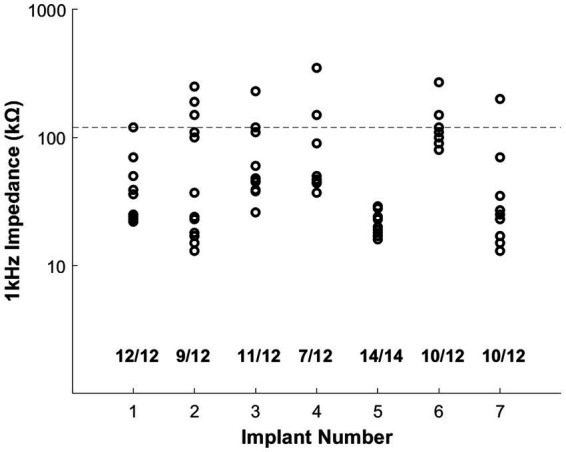
Impedance measurements immediately following initial array insertion for seven electrode implantations performed across four NHPs. Each marker represents the measured impedance for one electrode site. Values at the bottom of the graph indicate the proportion of electrodes suitable for stimulation (impedance below 120 kΩ, marked with a horizontal dashed line across the plot) after insertion. Each array had a total of 12 active sites, with the exception of implant 5 which had 15 active sites. For implant 5, one site was excluded due to a defect identified during the manufacturing process. Electrodes deemed to be completely broken or detached (impedance greater than 1 MΩ) were excluded from the plot but contributed to the total counts shown for each implant. The majority of electrode shanks (73/86) showed favorable impedance values ranging from 10 to 120 kΩ, highlighting the durability of the ANI array during the surgical manipulation and insertion process.

The electrode shanks on the ANI array proved durable during the array insertion process, as assessed through impedance measurements. The baseline impedance of all electrode sites as measured in saline prior to implantation was determined to be ≤ 10 kΩ. Upon insertion, impedances in the range of 5–120 kΩ were deemed to be usable for stimulation. To arrive at this range, we considered that this ANI is designed for future use with clinically available stimulators used with CIs, such as those produced by our industry partner MED-EL. The compliance limit of such stimulators for safe stimulation restricts the acceptable impedance of each electrode site to 140 kΩ. To allow for some margin of error in measurements, a conservative limit of 120 kΩ was used as a cutoff for acceptable electrode impedance for stimulation. Values greater than 1 MΩ were seen for shanks that were completely broken from the array base or cases in which excessive bending of the wire bundle caused some electrode wires to be detached from the bond pad of the array interface. Across seven implantations, an average of 85 ± 6% (*n* = 7, mean ± standard error) of electrode shanks on the array were deemed to have acceptable impedances (data from our first implantation was excluded due to damage to the device wire bundle, which prevented electrophysiological measurements). The mean impedance across all measured electrode sites (excluding 3 sites whose impedance was ≥ 1 MΩ) was 66.12 ± 7.17 kΩ (*n* = 83, mean ± standard error). Figure 5 summarizes the overall distribution of measured impedances at initial implantation for all procedures performed. A full summary of implantation outcomes across all animals is provided in Supplementary Figure 2. In some cases, several shanks exhibited higher impedances (e.g., implants 2 and 4), possibly due to the shank tips hitting against the bony backing of the IAC during insertion. We also observed variation in device robustness, with some devices having stronger and more durable shanks which were more resistant to breakage (further details provided in discussion).

### 3.2 Electrically-evoked auditory brainstem responses

**Figure 6:**
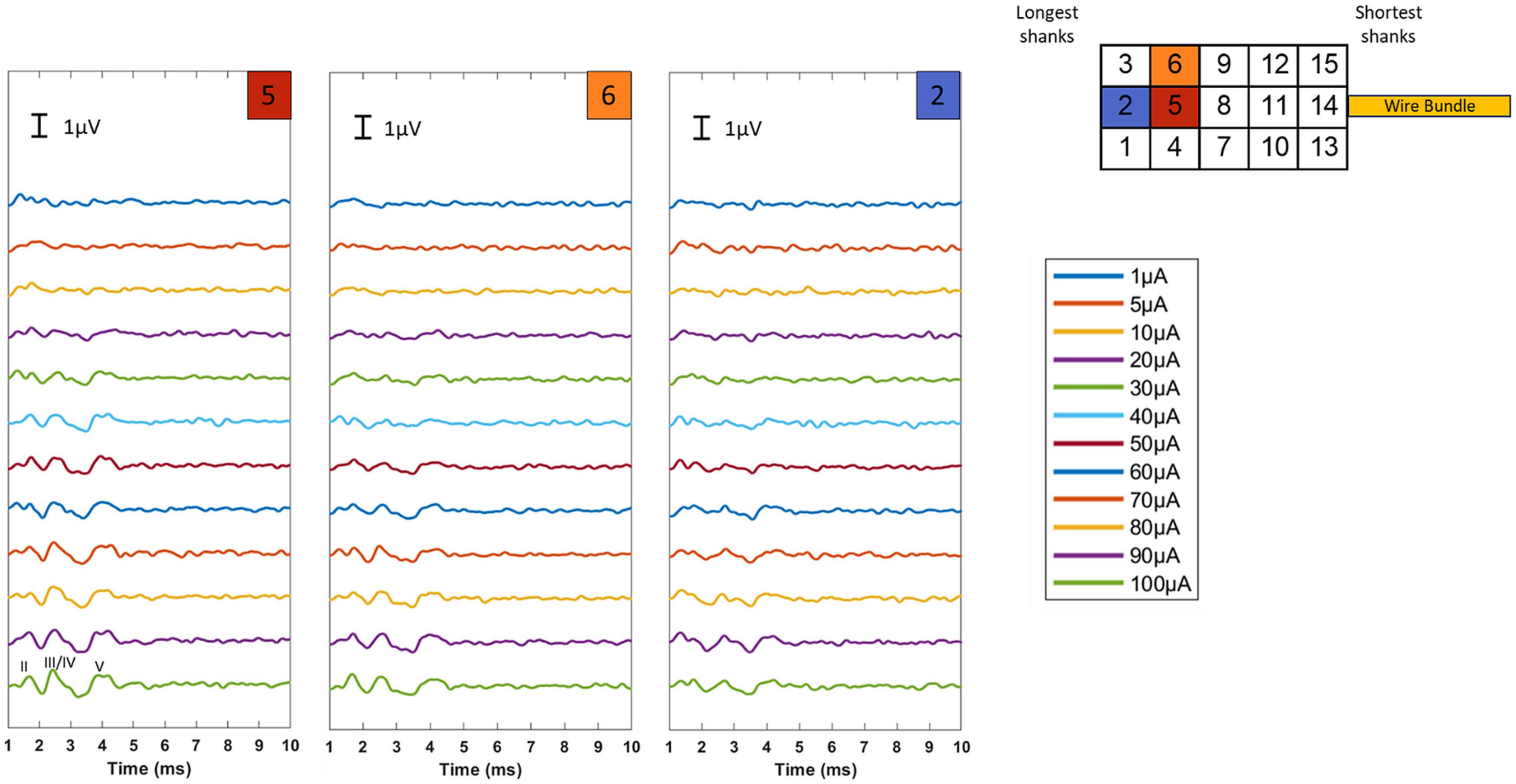
eABR level series for three different electrode sites for implant #1. The stimulation artifact (*t* = 0) has been blanked. Low thresholds (marked by the appearance of wave V) are seen for all three electrode sites, typically occurring between 10 and 50 μA. Roman numerals are used to denote the individual wave components of the ABR.

eABR datasets with responses on multiple electrodes were successfully collected for two implantations (implants 1 and 5 in Figure 5). The lack of observed responses during other implantations could be due to insufficient or incomplete insertion into the nerve (i.e., poor contact with nerve fibers), movement of the array out of the nerve after initial insertion, or potential damage to the nerve during the surgical exposure or implantation procedure. For implants 1 and 5, respectively, 8/12 sites and 6/7 electrode sites tested produced ABRs. Low activation thresholds (10–50 μA), characteristic of intraneural stimulation, confirmed direct contact of the electrode shank tips with auditory nerve fibers. ABR amplitudes were similar to those observed using traditional acoustic stimulation in past studies in the rhesus macaque (Kraus et al., 1985; Engle et al., 2013), ranging from 0.2–1.2 μV. Multiple component waves could be identified, indicating successful activation of downstream auditory structures (Waves I and in some cases II were obscured by the electrical artifact). Both polarities of the biphasic stimulus waveform (i.e., cathodic first versus anodic first) produced ABRs. Figure 6 shows the resultant ABRs for all stimulus levels tested for three example electrode sites. These results represent the average of both cathodic and anodic-leading stimulus waveforms, helping to verify that the response is of neural origin and not due to contamination by electrical artifact. Responses were similar in amplitude and waveform to acoustically-evoked ABRs collected prior to implantation (see Supplementary Figure 1 for examples).

**Figure 7:**
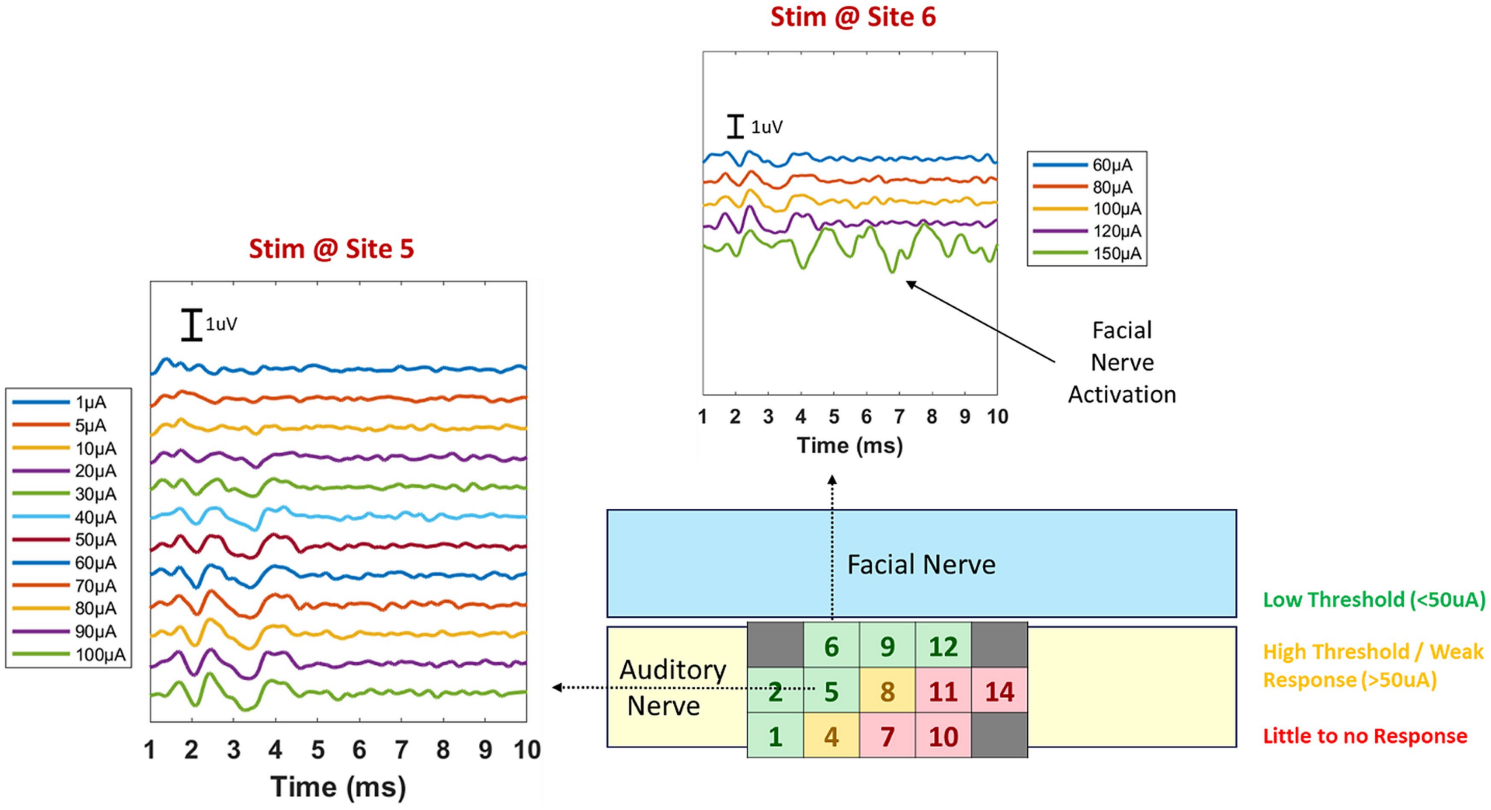
Example eABRs for implant #1 at two stimulation sites. One site (site 5) is located in the central row of the array while the other (site 6) is on a side column closer to the facial nerve. Because of the proximity of site 6 to the facial nerve, we sought to identify safe current limits for which activation was confined to the auditory nerve. Facial nerve activation was observed only when high current levels (150 μA) were employed. This result helped to verify that most current spread at reasonable stimulation levels (≤100 μA) was restricted to the auditory nerve itself and that off target activation was limited.

Amplitudes grew gradually as current levels were increased and saturated at higher current levels. Facial nerve activation was observed only when much higher current levels were employed (e.g., 150 μA), and only on electrode sites that were neighboring the facial nerve. Facial activation showed much higher amplitudes and an extended waveform with latencies much later than a typical ABR (Figure 7). The distribution of thresholds followed a spatial pattern somewhat in alignment with the length of electrode shanks along the array. Shanks that penetrated more deeply (e.g., sites 1, 2, 5, and 6) exhibited lower thresholds than shanks contacting more superficial areas of the nerve (sites 7, 10, and 11). It is possible that some shorter electrode shanks did not penetrate as fully as longer shanks and were potentially stimulating the surface of the nerve rather than the interior, thus requiring higher current levels to achieve activation thresholds. The more consistent responses at longer shanks could also be due to the top of the array (longest shanks side) being positioned under the bony crest, which helped those long shanks remain more firmly fixed within the nerve. Additional methods must be explored to place similar downward forces on the rest of the electrode array to ensure that the shorter shanks also penetrate the nerve fully.

**Figure 8:**
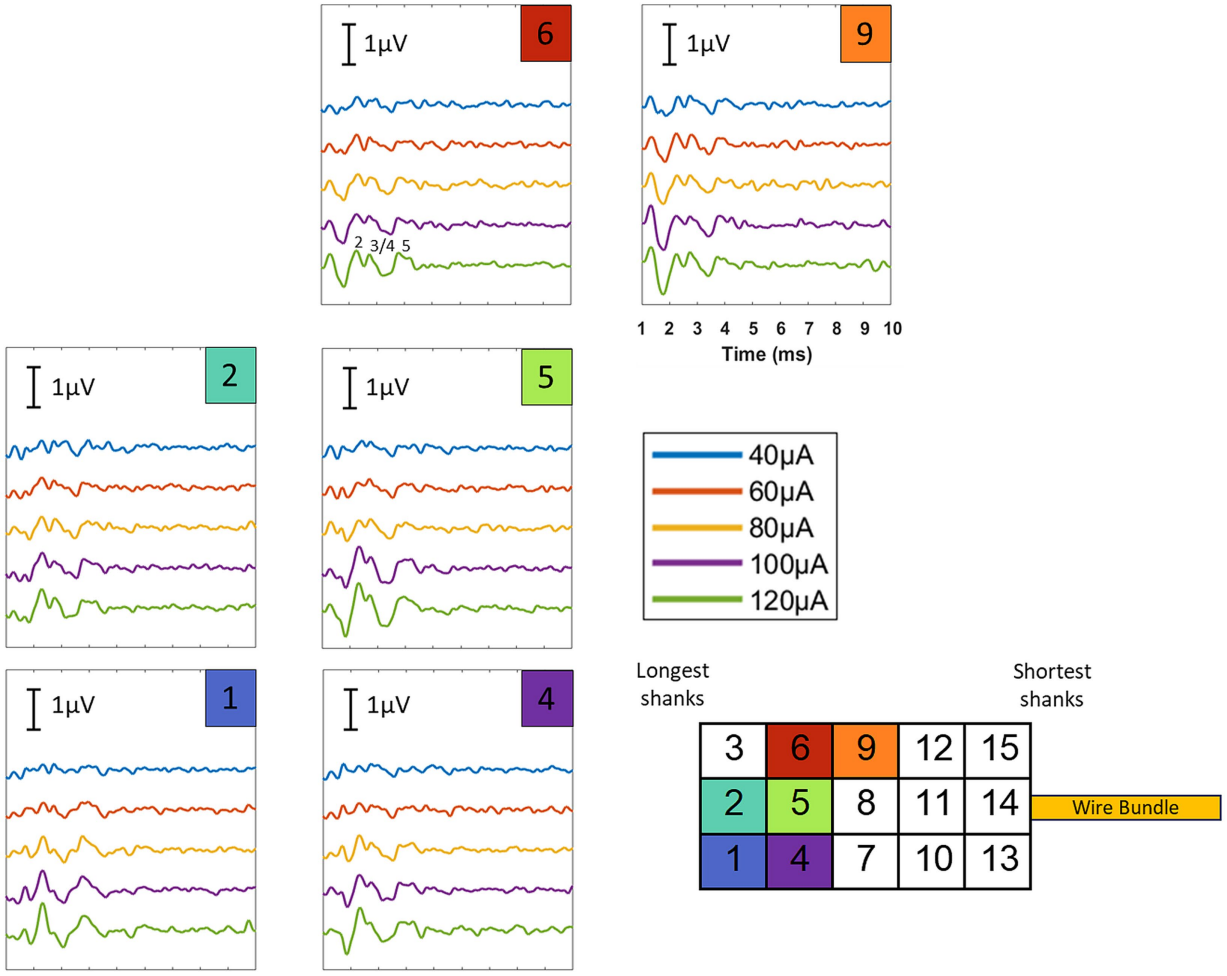
eABRs elicited from stimulation at 6 electrode sites for current levels ranging from 40 to 120 μA (results from implant #5). Differences in the ABR waveform morphology and amplitude were observed for different stimulation sites, which could indicate that some shanks penetrated more fully than others. Further work is required to characterize whether these differences could also be the result of frequency-specific activation by each stimulation site. It was confirmed that these waveform differences were not due to noise fluctuations or changes in the recording conditions over time (see Figure 9).

Variations in the ABR waveform morphology (Figure 8) were also observed depending on the electrode site that was activated. Because our electrode shanks likely penetrated to different degrees (i.e., shorter shanks in some cases did not penetrate fully), the differences in observed waveforms could be due to contacting different total amounts of auditory nerve fibers. Evidence of frequency-specific activation by each electrode site (which could also result in ABR waveform variations) will require follow up experiments which employ, for example, forward masking paradigms across different stimulation electrodes (Badi et al., 2006) or concurrent recordings in downstream tonotopically organized brain areas (Johnson et al., 2016).

**Figure 9:**
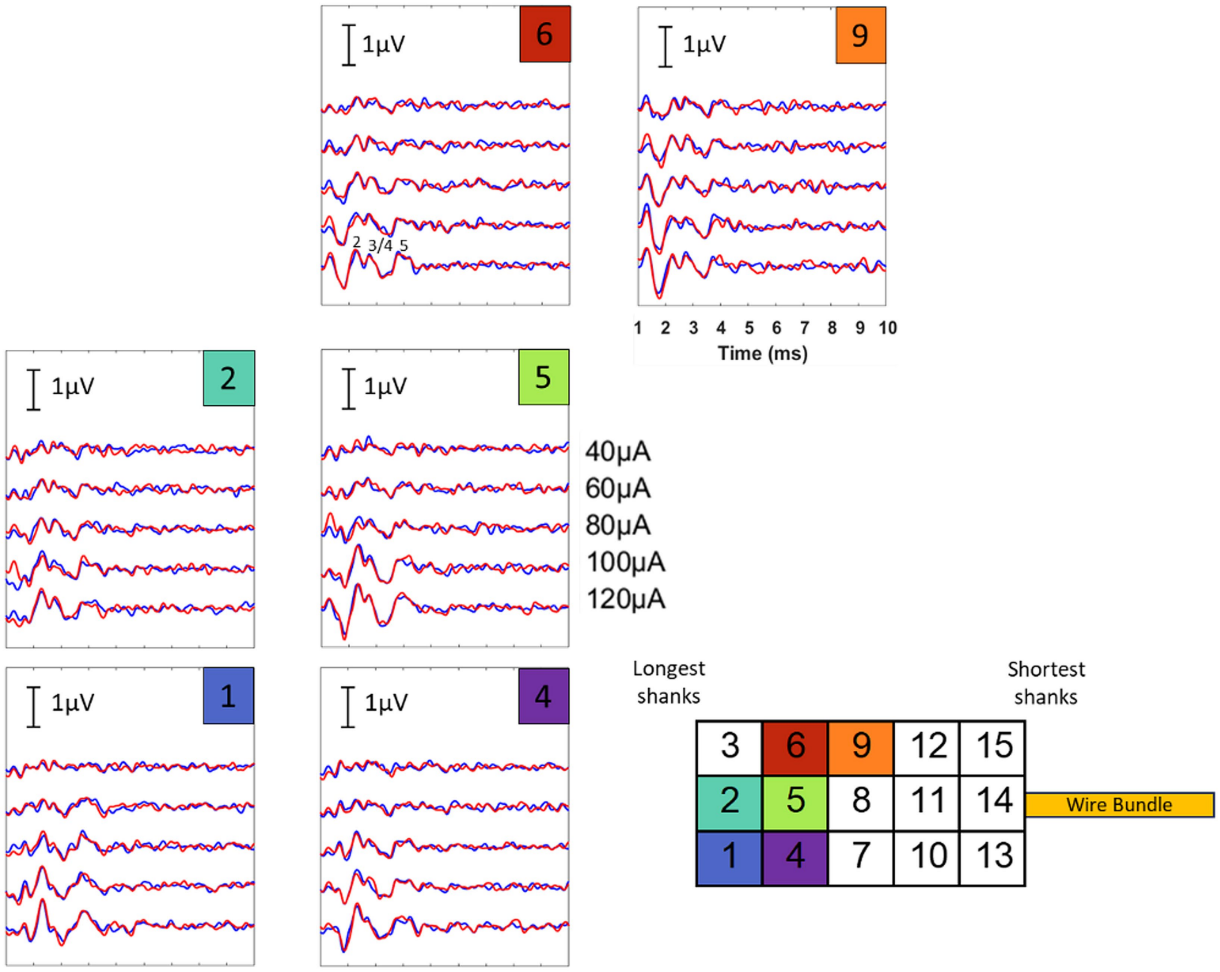
eABRs presented in Figure 8 replotted with trials from the first and second halves of the collection period analyzed separately. Blue traces show the averaged trials for the first half of the collection period while red traces show the averaged trials for the second half. Similar waveforms are seen for both halves of the collection period for each stimulation site, showing stability and consistency of the recording conditions and indicating that the waveform variation observed across sites is not simply due to noise fluctuations or changes in recording conditions over time.

### 3.3 Electrically-evoked compound action potentials

**Figure 10:**
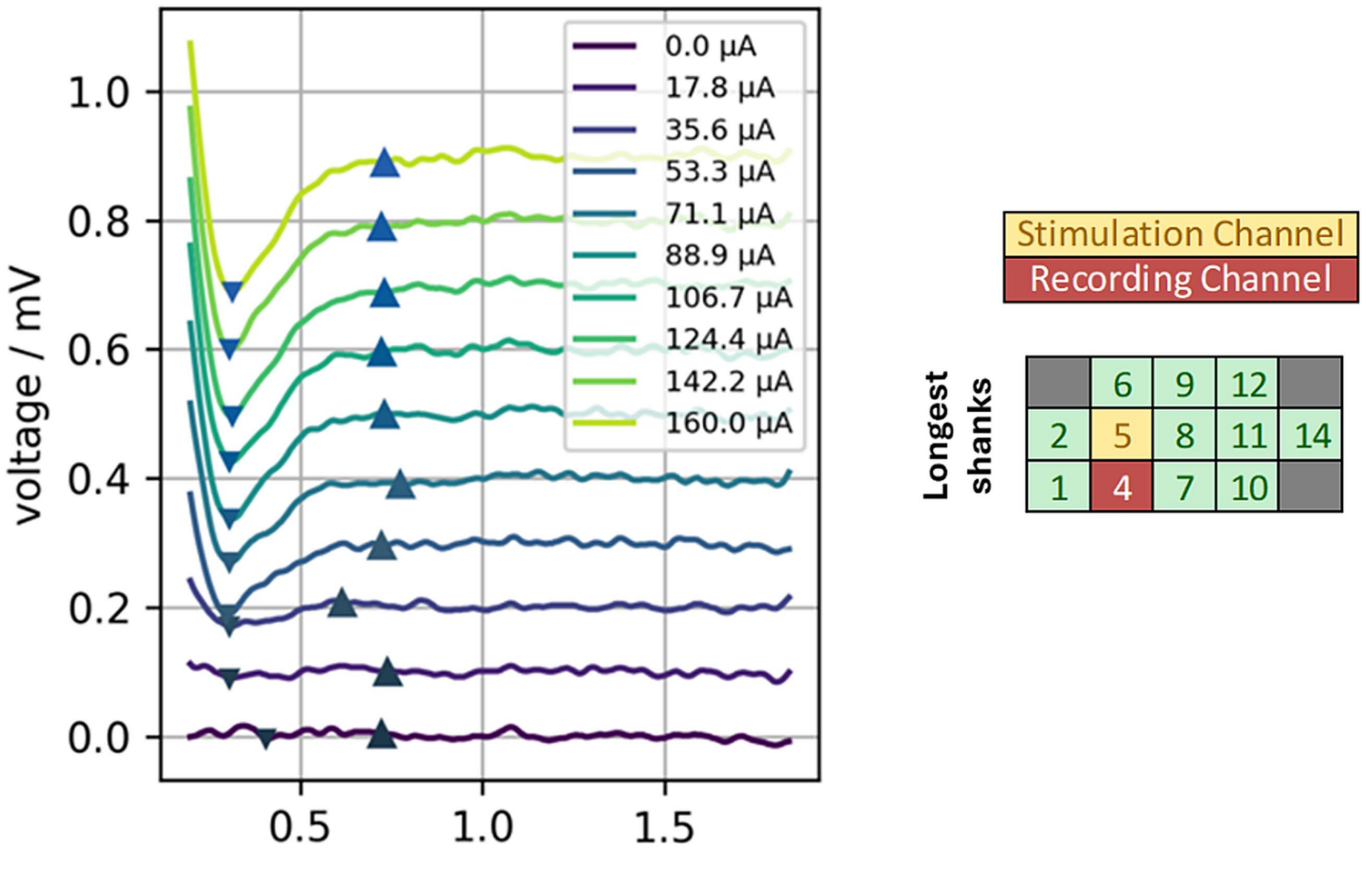
eCAP responses collected via stimulation of electrode site 5 and concurrent recording at neighboring electrode site 4 for implant #1. The eCAP amplitude grew as the current level was increased and began to saturate at ~124 μA. Saturation helped to confirm that the measured response was due to auditory activation and that activation did not spread to the facial or vestibular nerves. Up and down arrow indicators denote the positive and negative peaks (respectively) used to calculate response amplitude.

eCAPs were collected for three stimulation sites for implant #1. Similarly low activation thresholds (<50 μA) were seen for eCAPS as for ABRs (Figure 10). For each electrode site stimulated, the eCAP was recorded at four other sites on the electrode array. The examples shown here are for the recording site that gave the clearest eCAP response as determined through visual inspection of each channel. An example in which a range of currents were applied (0–160 uA) to a single electrode and the resulting waveforms recorded on a neighboring electrode is shown in Figure 10.

**Figure 11:**
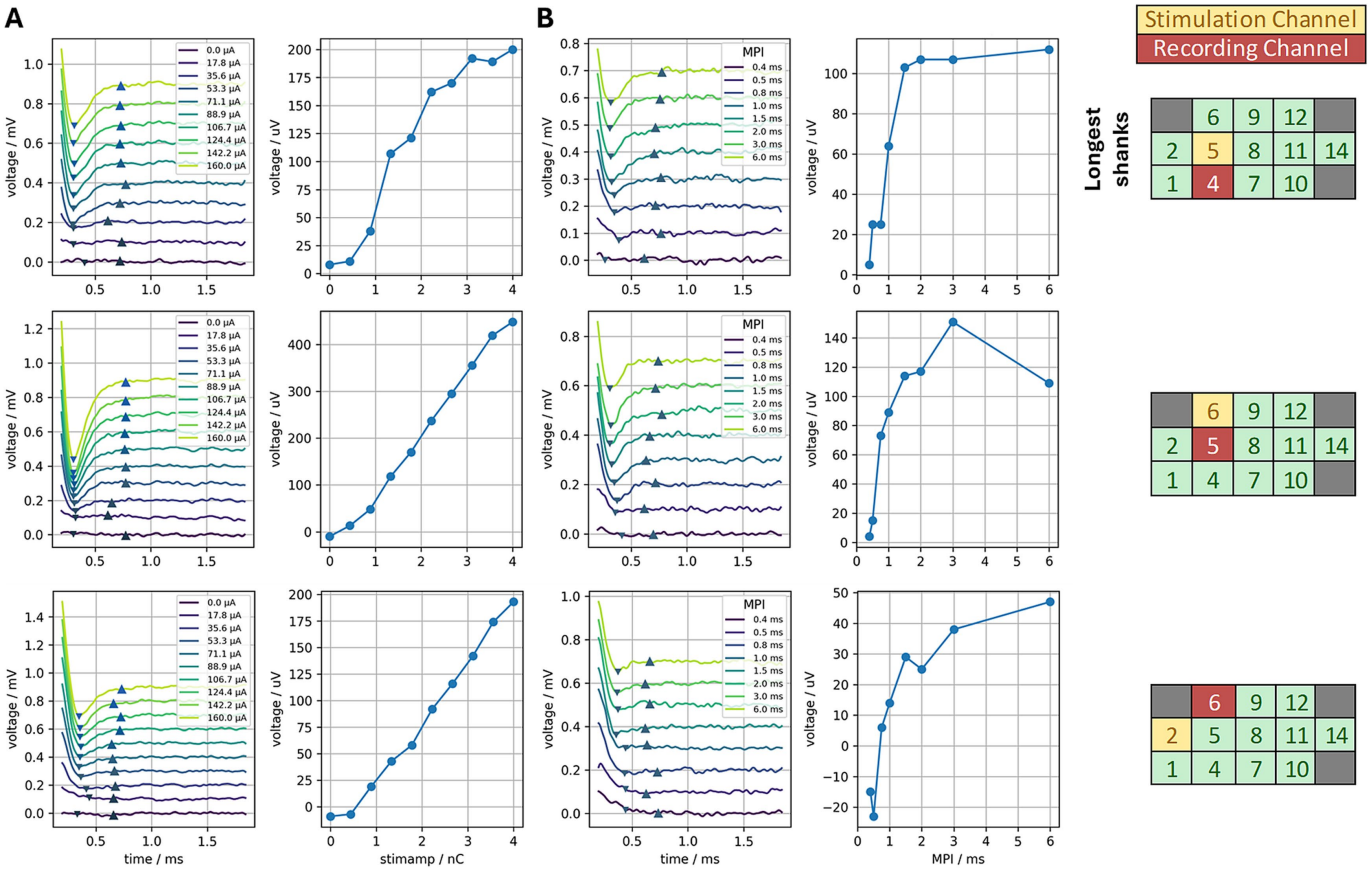
**(A)** eCAPs recorded for stimulation at three different electrode sites. Stimulation currents ranged from 0–160 μA. The left column shows averaged traces for each current level tested; the right column plots the amplitude growth function (amplitude of the eCAP as a function of stimulation amplitude in nano-Coulombs). **(B)** eCAPs recorded using a forward masking paradigm, in which two pulses are delivered with a varying masker-probe interval (MPI) and the eCAP measurement is performed following the 2^nd^ pulse. The left column shows the averaged eCAP traces for each MPI while the right column (recovery function) shows that as the MPI is decreased, the amplitude of the eCAP also decreases (i.e., lower MPIs give less recovery time for the auditory nerve fibers). When a 2^nd^ stimulation pulse is delivered soon after the 1^st^ pulse, some proportion of the auditory neurons are still in a refractory state from activation induced by the first pulse, thus leading to a smaller eCAP amplitude.

Amplitude growth functions in 1/3 cases (stimulation site 5) showed saturation at higher current levels (Figure 11A). Site 6 showed some continued growth even at the highest current level tested, possibly indicating that it could yet recruit a larger number of auditory nerve fibers or that some facial nerve activation occurred at higher levels and contributed to the main auditory response. The latter is likely the case, as facial nerve activation was observed during ABR recordings when site 6 was stimulated at 150 μA. Validity of the eCAP was established through generation of the recovery function (Figure 11B) via a masker-probe paradigm. eCAP amplitudes were significantly diminished for masker-probe intervals shorter than 1 ms, indicating that the neural elements generating the eCAP were still in a refractory period when the 2^nd^ pulse was delivered. This result supports that the observed response is due to neural activation and not simply measurement of the electrical artifact.

## 4 Discussion

The translabyrinthine approach allows for clear access and implantation of the NHP auditory nerve. Even accounting for anatomical variabilities across animals, in each case the vestibulocochlear nerve was identified and separated to isolate the auditory nerve. While our surgical approach involved sacrifice of the ipsilateral vestibular function, the deficiency was well accommodated. The electromechanical integrity of the array was also maintained during insertion, with the majority of electrode shanks remaining intact as evidenced through impedance readings. In some cases, low-threshold eABR and eCAP measurements verified that the electrode shank tips were placed very close to the stimulated auditory nerve fibers. These findings are in agreement with model-predicted activation thresholds for auditory nerve fibers, whereby the observed stimulation thresholds (10–50 μA) require that the electrode tip is placed approximately 40–200 μm away from the nearest node of Ranvier along the fiber (assuming a 0.1 ms cathodic stimulation pulse) (Rattay, 1987). Off-target activation of the facial nerve was limited, only occurring at high current levels that would not otherwise be used in typical clinical settings. The inconsistency in elicitation of eABRs across animals is likely due to incomplete penetration of the electrode shanks into the auditory nerve or movement of the array out of the nerve after insertion due to tension in the wire bundle and the large dimensions of the array relative to the NHP anatomy. It is also possible that damage to the nerve or nerve ischemia could have occurred during array insertion or surgery. To better characterize potential damage caused by ANI surgery or implantation, further studies such as post-mortem histological analysis can be performed to confirm full penetration of the electrode shanks into the nerve, characterize nerve integrity, and assess the tissue response. As done in prior studies, the temporal bone can be extracted, fixed with formalin solution, and embedded in a resin matrix (Rousselle et al., 2019; Rau et al., 2013). A laser microtome can then be used to section the nerve sample without removal of the implant (LLS Rowiak, 2025), which allows for accurate depiction of electrode shank penetration depth and also prevents damage to the nerve resulting from device explantation. High resolution imaging can give insights into potential damage through inspection of the cochlear axons and cell bodies. Immunohistochemistry techniques can also be employed to highlight the local tissue response and quantify the extent of inflammation (Lopez et al., 2016).

The challenges with electrode insertion were largely due to the electrode array used in this study (designed for human use) being large compared to the macaque anatomy. These types of issues were not seen during preliminary intraoperative surgeries performed in humans, in which the surgical cavity is larger and more easily accommodates the ANI assembly (Lenarz, 2023; Adams and Lenarz, 2023). In those cases, we were able to position the device and wire bundle in a more stable configuration and confirm successful array insertion with impedance and eABR recordings (publication in progress). We anticipate conditions for stability will be more favorable in humans than in the model used in this study. Our findings show viability of the NHP for continued auditory nerve implant studies, given a modified device designed for the rhesus monkey and that improved methods are developed for ensuring full and stable insertion of electrode shanks into the nerve. In terms of device design, a reduced number of electrode shanks, alternative shank configurations (e.g. 2 columns of shanks rather than 3 and with multiple electrode sites placed along each shank rather than only at the tip), and/or a smaller inter-electrode distance would provide a better size match of the array with the rhesus macaque auditory nerve and thus facilitate array positioning and insertion. We also observed in our eABR data that the shortest shanks required much higher activation thresholds or failed to elicit eABRs at all, suggesting that they possibly contacted only the surface of the nerve or that the shank tips were outside the nerve altogether. We believe this was attributed to the inability to fully insert the array into the auditory nerve due to its large size and excess tension in the wire bundle, which should also be addressed with the device improvements presented above.

Anatomic variability across animals also played a role in implantation outcomes. A major source of this variability was the size of the mastoid cavity, which was bound by the sigmoid sinus and posterior fossa dura. In cases where the cavity was smaller, stable placement and fixation of the device was more challenging. Furthermore, blood supply patterns to the cochlear nerve could have also differed in each animal. If so, reflection of the vestibular nerve may have reduced the blood supply to the cochlear nerve more so in some cases than in others. In addition to anatomic variability, we also note that as a developing technology, the USEA used in this study underwent continual improvements in its design and manufacturing processes throughout the study period. Due to manufacturing variability, some USEAs exhibited sharper electrode tips (making insertion easier in some cases) and more durable shanks that were less prone to breakage or cracking. Our study has helped to improve the device manufacturing processes, thus current devices have become reliable and consistent in their mechanical characteristics.

Further work is required to establish feasibility of the macaque as a chronic model. As we observed during multiple initial insertion procedures and during acute stimulation sessions, the array shanks and electrode sites shifted out of the nerve, which was largely attributed to the excessive tension in the wire bundle caused by the sharp bends required to fit the large array into the small bone cavity in the NHP. Fat grafts were used to pack the surgical space and place pressure on the array to keep it inserted in the nerve. A bony outcropping was also identified (transverse crest in Figure 4B) under which the array could be placed before full insertion in some animals. This bony covering helped to apply pressure on the array and counteract fluid pulsations. For future chronic implantations, additional methods to secure array positioning should be explored, in addition to matching the dimensions of the array and design of the wire bundle more accurately to the size and anatomy of the NHP. Changing the connection type between the implant and stimulation system could also improve stability and ease of implantation. A percutaneous connector (secured to the outside of the skull of the animal) was used for the current study, to which a wired connection to the stimulator was established during experimental sessions. Maneuvering and placement of the percutaneous connector during the surgical procedure greatly increased the difficulty of implantation. Repeated connection to the connector for each stimulation session could also cause strain and risk of array or wire displacement. Furthermore, while a protective cover was placed on the percutaneous connector when not in use, manipulation and picking at the connector site by the animal subject could cause residue and debris to build up on the edges or surfaces of the connector area if the protective covering is breached or damaged over time. For this reason, we envision that the future connector type for this ANI prototype will involve a magnetic coil similar to that used by modern CIs, thus allowing for wireless transmission of power and stimulation signals while avoiding connector damage and/or strain on the array wire bundle.

If stability and consistent insertion of the array can be maintained, avenues open for studies of chronic implantation. Such studies could be useful not only for testing of device stability and durability, but also to corroborate electrophysiological data with behavioral experiments. Past studies have successfully engaged NHPs to perform frequency discrimination tasks (Sinnott and Petersen, 1983; Pages et al., 2016; Joly et al., 2014) and other psychoacoustic tests of auditory perception (Jonathan, 1974; Lemus et al., 2009; Shyan et al., 1987). Similar to discriminating tones of different frequency, these tasks could be performed using electrical stimulation of different regions of the auditory nerve to ascertain frequency-specific activation. To perform such experiments, it is critical that the array remains in a stable and consistent position throughout the study period. Even minor shifts in array position could change the frequency fiber groups that each electrode shank targets.

The successful recording of eCAPs using this ANI device shows promise for the auditory nerve eCAP as a tool to verify proper electrode placement and ascertain nerve health, not only in animal models but also in future human ANI patients. The convenience of being able to stimulate and record on the same electrode array prevents the need for additional recording systems involving placement of needle or EEG electrodes, which saves time and reduces complexity in a surgical setting. Following implantation, the eCAP amplitude and spread of excitation for each electrode site can be regularly recorded over time to track stable device positioning. Changes in amplitude or spread of activation could be used to indicate displacement or movement of the array (Berg et al., 2024). This would allow for early detection of complications and/or initiation of follow-up correction surgeries/procedures if needed. In future studies, the multi-site eCAP (i.e., in response to low current simultaneous stimulation at multiple electrodes) could be used to quantify how precisely each electrode is stimulating different sections of the nerve. Forward masking experiments, similar to those performed with CI arrays (Tang et al., 2011), can also be used to determine the amount of overlap between the activation fields of different electrode sites. These measurements can give insights into the channel interactions between electrode sites and allow estimation of stimulation specificity.

## 5 Conclusion

The NHP auditory nerve can be accessed and implanted via the translabyrinthine approach without noticeable damage to the nearby facial nerve. Impact to the overall health of the subject is minimal and vestibular deficits are typically compensated. Electrophysiological measurements confirm that direct intraneural stimulation can be achieved using the electrode array presented here without significant current spread to the surrounding area. However, future studies involving chronic implantation must focus on improving the design and dimensions of the ANI assembly for the size and anatomy of the NHP that can improve array insertion and stability of electrode sites within the auditory nerve. With further refinement of this surgical approach and implantation methods, the rhesus macaque model has potential as a preclinical testbed for future ANI technology. In addition to electrophysiological studies, behavioral and psychoacoustic experiments could also be performed in the NHP, giving new insights into perceptual aspects of intraneural stimulation for developing novel stimulation strategies.

## Data Availability

ABR data were uploaded to the Data Archive for the BRAIN Initiative (DABI) repository. The data can be found under the project code UG3NS107688.
