# Supplementary material for: Development of a non-human primate model for preclinical research of a novel auditory nerve implant
Source: Front Neurosci. 2025 Dec 10;19:1669116. doi: 10.3389/fnins.2025.1669116 (PMC12728018; doi:10.3389/fnins.2025.1669116)
Supplement: Supplementary file 1 [file Data_Sheet_1.docx]

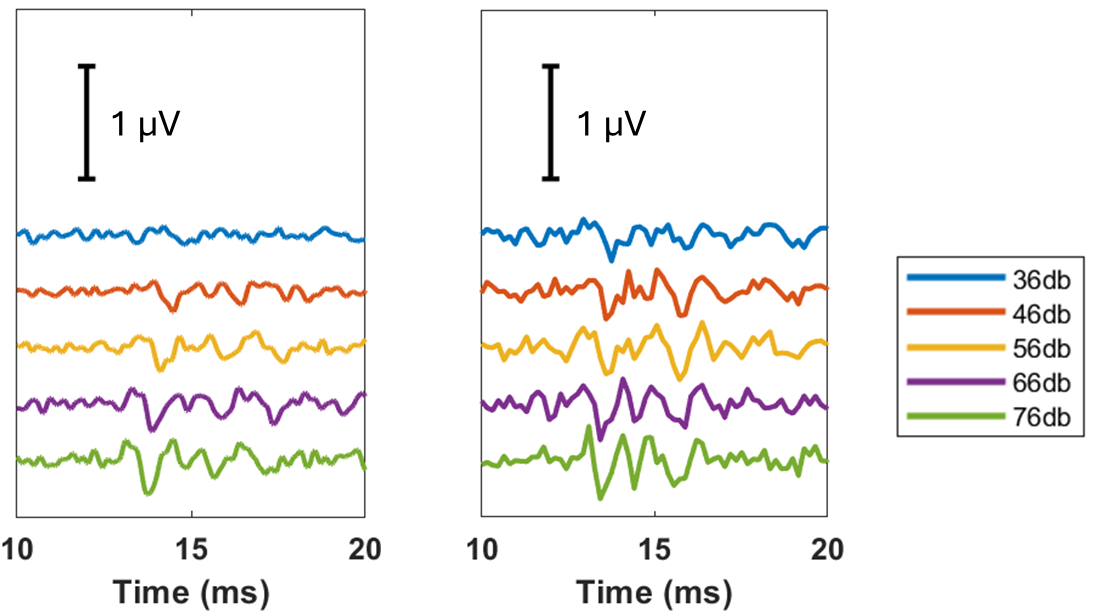


**Supplementary Figure 1.** ABRs collected in response to acoustic stimulation (10 ms noise burst at intensities ranging from 36-76 dB SPL) in two example NHPs prior to ANI implantation. Multiple waves of the ABR are observable, with an increase in peak amplitude and decrease in peak latency as the sound intensity level is increased. These results demonstrate typical hearing levels in the animals before undergoing the translabyrinthine surgical procedure.

**Supplementary Figure 2.** Summary of implantation outcomes across all animals. In all implantations, the majority of electrode shanks remained intact as indicated by low impedance values. eABRs were successfully detected in 2/7 implantations. No significant surgical complications (e.g. facial side effects, meningitis, CSF leak, etc.) occurred during any of the implantation procedures.
